# Effects of Sesamin on Streptozotocin (STZ)-Induced NIT-1 Pancreatic β-Cell Damage

**DOI:** 10.3390/ijms131216961

**Published:** 2012-12-11

**Authors:** Hong Lei, Juncheng Han, Qin Wang, Shuzhen Guo, Hanju Sun, Xiaoxiang Zhang

**Affiliations:** School of Biology and food engineering, Hefei University of Technology, Hefei 230009, China; E-Mails: hanjuncheng88@163.com (J.H.); zhongqin321@hotmail.com (Q.W.); guoszhen@126.com (S.G.); sunhanjv@163.com (H.S.); emailzxx@yahoo.com.cn (X.Z.)

**Keywords:** sesamin (SES), NIT-1 pancreatic β-cell, streptozotocin (STZ), protective effect

## Abstract

The protective effect of sesamin (SES) from sesame meal on NIT-1 pancreatic β-cells damaged by streptozotocin (STZ) *in vitro* was investigated. The cell viability, insulin secretion, the activity of superoxide dismutase(SOD), glutathione peroxidase (GSHpx) and the content of reduced glutathione (GSH) increased significantly when incubated with SES (400, 200 μg mL^−1^). The content of malondialdehyde (MDA), nitric oxide (NO) production, and the activity of NO synthase (NOS), inducible NOS (iNOS), decreased significantly when incubated with SES. The destructive changes of NIT-1 cells were ameliorated when treated with SES under microscopic observation. These data suggested that SES had obvious protective effect on NIT-1 pancreatic β-cells damaged by STZ, which might be related to its effects of decreasing levels of β-cell-destroying factors such as oxidative stress and NO synthesis.

## 1. Introduction

Sesame seeds and oil have been praised and consumed by Chinese people for thousands of years. Its active part is sesamin (SES), which is one of the most abundant lignans in sesame and has multiple functions and high values [1]. Sesamin can be extracted from sesame seeds, sesame oil, and sesame meal [2]. Moreover, the medicinal properties of sesamin have been demonstrated scientifically and experimentally. For instance, sesamin has been shown to have anti-oxidative effect [3–5], promoting-immunity function [6], anti-carcinogen activity [7,8], blood pressure-lowering effect [9], serum lipid-lowering and hepatocyte-protecting effects [10–15].

Sesamin has been extracted and purified from sesame meal in our laboratory. Previous studies have shown that sesamin has hypoglycemic activity in KK-Ay mice, a kind of type 2 diabetic animal model, by ameliorating peripheral insulin resistance *in vivo*. In order to evaluate the ameliorating effect of sesamin on diabetes comprehensively, the present study was therefore designed to explore the protective effect of sesamin on NIT-1 pancreatic β-cells damaged by streptozotocin (STZ) *in vitro*. Moreover, its mechanisms of action concerning protection of pancreatic β cells from destruction by oxidative stress and nitric oxide (NO) *in vitro* were also investigated.

## 2. Results and Discussion

### 2.1. Purity of Sesamin (SES) Sample

**Figure 1:**
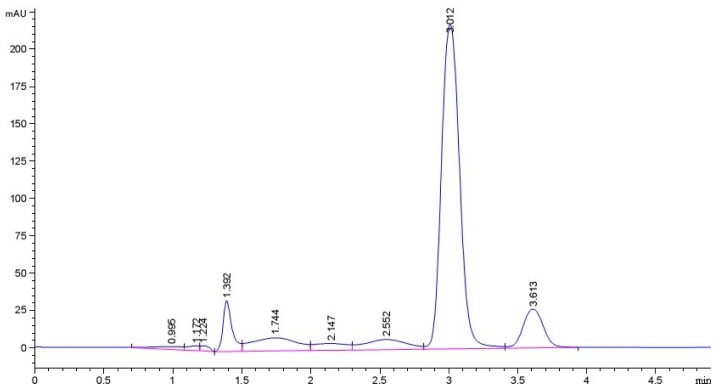
The HPLC diagram of sesamin sample.

The linear regression equation is *Y* = 14.82*X* + 1.737 (*R*^2^ = 0.9995), in which *X* represents concentration of sesamin standard and *Y* represents absorption peak area (mAU). Figure 1 shows the HPLC diagram of sesamin sample which has a relatively symmetric peak at the retention time of 3.012 min. The purity of sesamin sample is 70.56% which was calculated according to the linear regression equation.

### 2.2. Effect of Cytotoxicity of Streptozotocin (STZ) on the Pancreatic NIT-1 Cells

The NIT-1 cell line was established from the insulinomas that developed in the transgenic NOD mice, and the cells were transformed with a hybrid rat insulin promoter/SV40 large T-antigen. Preliminary work on the NIT-1 cell line showed that it possessed many characteristics and ultrastructural features of normal differentiated mouse pancreatic β cells, such as well-developed rough endoplasmic reticulum, extensive golgi apparatus and beta granules [16]. NIT-1 cells provide a substantial supply of immortalized β cells, which are normally difficult to obtain.

**Figure 2:**
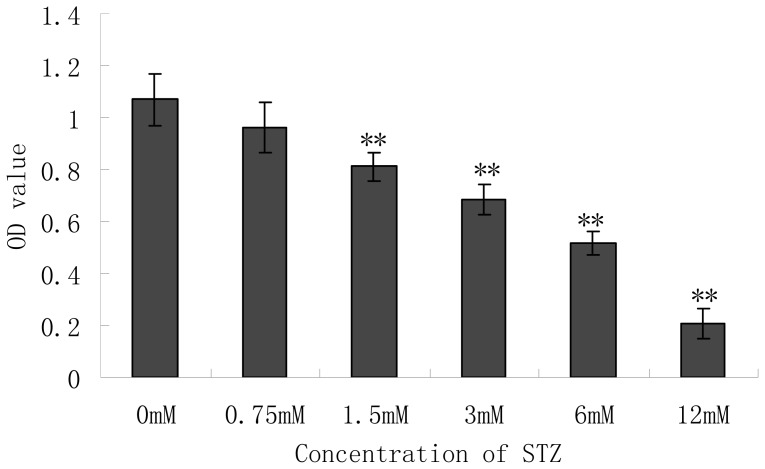
Effect of different concentrations of STZ on the NIT-1 cell viability. Data are the mean ± SD (*n* = 10). ^**^p < 0.01 compared with 0 mM control group.

Steptozotocin (STZ), an antibiotic produced by *Streptomyces achromogenes*, has the β-cell cytotoxic effect and is a kind of commonly used agent in experimental diabetes. In STZ-induced diabetes, hyperglycemia and β-cell destruction have been implicated in the etiology and pathology of diabetes [17]. Although the mechanism of β-cell cytotoxic action of STZ is not fully established, it is thought to act through oxidative damage, mediated by the inhibition of free radical scavenger-enzymes, thereby enhancing the production of the superoxide radical and NO. Chemicals with antioxidant properties and free radical scavengers were shown to prevent pancreatic islets against cytotoxic effects of STZ [18]. NIT-1 β cells were MTT assayed for viability which was expressed as the value of O.D. at 570 nm. The effect of different concentrations of STZ on the viability of NIT-1 cells was shown in Figure 2. *In vitro* administration of STZ to the NIT-1 cells for 24 h caused dose-dependent toxicity. Viability of NIT-1 cells reduced to a greater extent. STZ had a significant adverse effect on cells from the concentration of 1.5 mM compared with the untreated group (*p* < 0.01). At 6 mM of STZ, nearly 50% of the cells were necrotic and at the concentration of 12 mM, most of the cells (80%) were necrotic. We selected 6 mM of STZ to do the further experiment.

### 2.3. Effect of SES on the Viability of NIT-1 Cells Damaged by STZ

**Figure 3:**
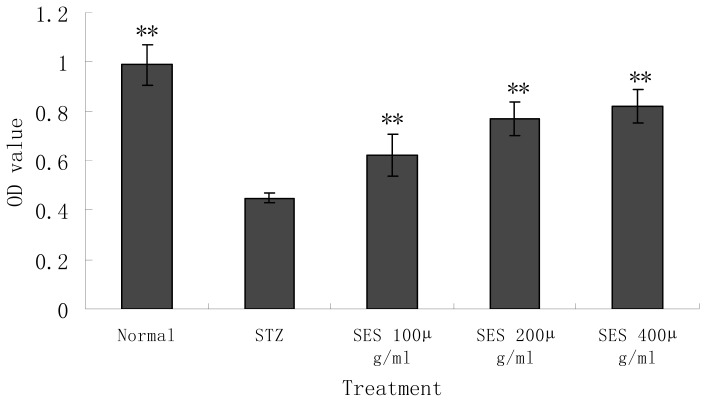
Effect of SES on the NIT-1 cell viability damaged by STZ. Data are the mean ± SD (*n* = 10). ^**^p < 0.01 compared with STZ model control group.

In the present study, we adopted the STZ-induced NIT-1 cell-injury model to investigate the protective effect of SES on the β cells *in vitro*. As shown in Figure 3, cell viability significantly decreased in the STZ-model control group compared with the normal control (*p* < 0.01). Administration of SES (400, 200 μg/mL) for 24 h significantly reversed STZ-induced cells’ viability loss; the O.D. values significantly increased (*p* < 0.01). NIT-1 cells destroyed by STZ showed significantly higher viabilities co-incubation with SES. However, co-incubation with SES (400 μg/mL) had no effect on the viability of NIT-1 cells without STZ (the OD value was 0.988 ± 0.083 *vs.* 0.954 ± 0.077, for normal control and SES-treated normal NIT-1 cell groups, respectively; as compared with normal control, *p* > 0.05). This indicated that treatment with SES significantly protected the NIT-1 cells from STZ-induced cell death, but had no effect on normal NIT-1 cells.

### 2.4. Effect of SES on Insulin Secretion by NIT-1 Cells Damaged by STZ

**Figure 4:**
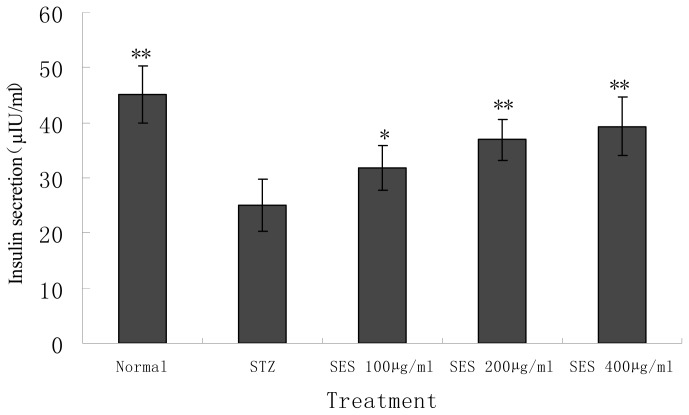
Effect of SES on insulin secretion by NIT-1 cells damaged by STZ. Data are the mean ± SD (*n* = 10). ^*^p < 0.05 and ^**^p < 0.01 compared with STZ model control group.

As shown in Figure 4, STZ had an adverse effect on insulin secretion by NIT-1 cells. Insulin production of STZ-treated cells decreased significantly compared with the normal control (*p* < 0.01). SES (400, 200, 100 μg/mL) exerted significant promoting actions on insulin secretion by NIT-1 β-cell compared with the STZ model control (*p* < 0.05 or 0.01). This suggested that SES has the improving effect on insulin secretion function of NIT-1 cells, which may be the consequence of protection of the cells damaged by STZ.

### 2.5. Effect of SES on Levels of SOD, GSHpx, GSH and MDA in NIT-1 Cells

*In vitro* and *in vivo* studies have suggested the implication of oxidative stress in the progression of β-cell dysfunction in type 2 diabetes [19]. It has been generally accepted that the pancreas is especially susceptible to STZ-induced free radical damage and low levels of key enzymes scavenging oxygen free radicals. Recently, we reported that SES has the anti-oxidative effect on KKay mice, a kind of type 2 diabetes animal model. In the present study, we investigate the effects of SES on the antioxidative activity of NIT-1 cells damaged by STZ *in vitro*. In order to determine the changes of the cellular antioxidant defense system, antioxidant enzymes such as GSH_Px_, SOD activities, and antioxidative molecule GSH content were measured. The changes of the levels of SOD, GSHpx, GSH and MDA of normal, model and SES groups were shown in Table 1. Results showed that the activity of T-SOD, GSHpx and the content of GSH of STZ-treated NIT-1 cellular model markedly decreased compared with the normal control (*p* < 0.05 or 0.01). The content of MDA markedly increased compared with the normal control (*p* < 0.01). After 24 h treatment with SES (400, 200 μg mL^−1^), T-SOD, GSHpx activity and GSH content markedly increased, MDA content markedly decreased in model group compared with the normal control group (*p* < 0.05 or 0.01). STZ induced the increased lipid per-oxidation and the decreased antioxidant enzyme activity significantly. Treatment with SES could reduce the content of the lipid per-oxidation product MDA of cells, and increase the activity of the anti-oxidative defense system and thus inhibit free radical generation. This suggested that SES has the anti-oxidative effect, which prevents and protects STZ-induced oxidative stress and β-cell damage.

### 2.6. Effect of SES on Levels of Nitric Oxide (NO), NOS, iNOS in NIT-1 Cells

It has been known that NO is involved in the pathogenesis of diabetes and the functional impairment of islet β cells and insulin production [20]. In addition to the macrophages which may be the major source of NO production in the process of islet inflammation, β-cells themselves have been shown to induce NOS and generate NO [21]. In the present study we investigated whether SES may protect NIT-1 β-cells from the destructive actions of NO induced by STZ *in vitro.*

The levels of NO, NOS and iNOS were shown in Table 2. The levels of NO, NOS and iNOS were significantly higher in STZ-treated NIT-1 cells as compared with normal control (*p* < 0.05 or *p* < 0.01). STZ might lead to the destruction of the pancreatic β cells by induction of increased expression of NOS and iNOS. Incubation of NIT-1 cells with SES (400, 200, 100 μg mL^−1^) lowered the NO, NOS, iNOS levels markedly as compared with untreated model (*p* < 0.05 or *p* < 0.01). Our results demonstrated that SES prevented the generation of NO by NIT-1 cells induced by STZ, and could inhibit the NOS, iNOS activity of pancreatic β-cells *in vitro*. This suggested that SES has the effect of suppressing the generation of NO, which is another β-cell destructive factor, and exerts β-cell-protecting effect.

### 2.7. Effect of SES on the Microscopic Observation of NIT-1 Cells Damaged by STZ

**Figure 5:**
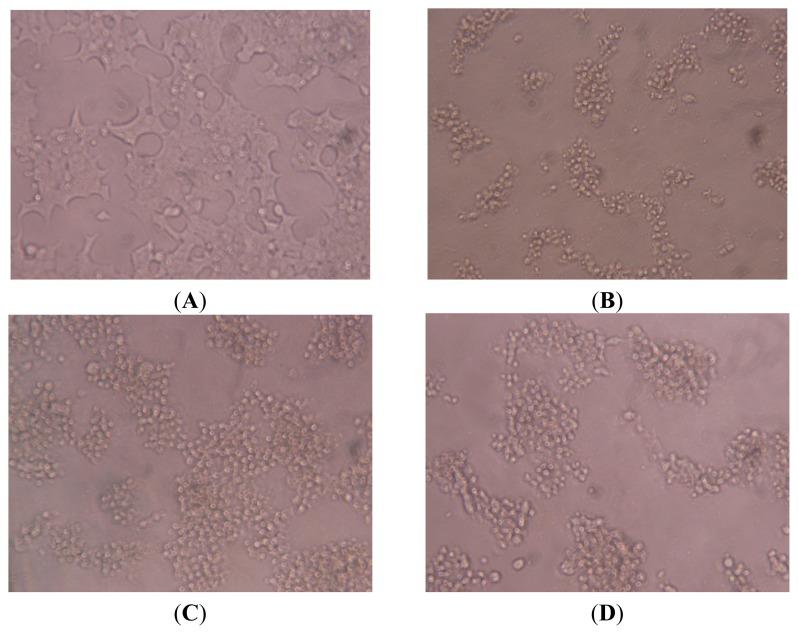
Effect of SES on microscopic observation of NIT-1 cells. (**A**) Normal control group; (**B**) STZ model group; (**C**) SES 400 μg mL^−1^ group; (**D**) SES 200 μg mL^−1^ group.

Healthy NIT-1 cells were observed under microscope (Figure 5A). The shape of normal NIT-1 cells was irregular polygon, adherent to the plate-wall in cluster aggregation. When incubating with STZ, the number of NIT-1 cells markedly decreased, and the shape—markedly pathological—underwent changes such as cell shrinkage and the development of a dark appearance and black-spots, as seen under microscope. Moreover, STZ also had a greater effect in reducing confluence of the NIT-1 cells (Figure 5B). This suggested that the damaged β-cellular model induced by STZ was successfully established. Treatment with SES (400, 200 μg mL^−1^) markedly restored the shape and structural integrity of the damaged cells as compared with model control (Figure 5C,D). This suggested that the pancreatic NIT-1 β-cells were obviously destroyed by STZ, while SES could ameliorate NIT-1 β-cell destruction significantly and had a β-cell-protecting effect.

## 3. Experimental Section

### 3.1. Preparation and Purity of Sesamin (SES)

Sesamin was extracted and purified from sesame meal. Dried sesame meal powder was pulverized and refluxed with ethanol (1:8.5 (g/mL)) at 55 °C for 2.5 h. After filtration, the filtrate was collected and the filter residue was refluxed with ethanol (1:8.5 (g/mL)) at 55 °C for 2.5 h again. After filtration, the primo-secondary filtrate was merged and collected and then was purified by macro-porous resin AB-8. The desorption liquid is ethyl alcohol and the liquid amount is 10 times the volume of the column (BV) with the elution velocity 1.0 mL/min. Then the eluent was collected, concentrated, and centrifuged at 2800 × *g* for 10 min. The sediment was collected, vacuum dried and the sesamin sample was obtained.

The purity of the sesamin sample was estimated by high-performance liquid chromatography (HPLC). HPLC was performed using Agilent 1260 series HPLC pump equipped with a Zorbax column (C18 4.6 × 100 mm 3.5 μm), using methyl alcohol and distilled water (8:2 (*v*/*v*)) as a mobile phase. The column temperature was 25 °C, the flow rate is 0.8 mL min^−1^ and the detective wavelength is 290 nm. The calibration curve of sesamin standard was drawn and the linear regression equation was established by linear regression method. The purity of sesamin sample was estimated and calculated based on the calibration curve made by HPLC using sesamin standard. Then sesamin sample was dissolved in DMEM medium and diluted to the concentration needed.

### 3.2. Main Reagents

Dulbecco’s modified Eagle’s medium (DMEM) was obtained from Gibco. Streptozotocin (STZ) and MTT were from Sigma. Trypsin was from Invitrogen Life Technologies. Various measuring kits were used during the study. These were as follows: insulin analysis kit (Shanghai Yuanxiang Medical Instrument Co., Ltd, Shanghai, China); superoxide dismutase (SOD), glutathione peroxidase (GSHpx), reduced glutathione (GSH) and malondialdehyde (MDA) measurement kit, nitric oxide (NO), NO synthase (NOS), inducible NO synthase (iNOS) measurement kit (Nanjing Jiancheng Bioengineering Institute, Nanjing, China). All the other biochemicals and chemicals used in the experiment were of analytical grade.

### 3.3. NIT-1 Cell Culture

The pancreatic β-cell line NIT-1 was purchased from ATCC (NO. CRL-2055) and cultured in Complete Dulbecco Minimum Essential Medium (DMEM) supplemented with 25 mM glucose, 15 mM HEPES, 1 mM sodium pyruvate, 2 mM L-glutamine, 2 g L^−1^ sodium bicarbonate, 100 mg L^−1^ Penicillin/Streptomycin, 10% heat-inactivated Fetal Calf Serum (FCS) which were adjusted to pH 7.2, and maintained in 75 cm^2^ tissue culture flasks at 37 °C in a humidified atmosphere of 5% CO_2_ incubators. Cells were allowed to attach to the flask. Cell culture medium was exchanged every 48 h and cells were passaged at weekly intervals by trypsination. Harvesting and passaging of the NIT-1 cells were accomplished by detaching, aspirating and separating the adherent cells by mechanical agitation, followed by incubation with 0.25% trypsin and 0.02% EDTA in D-Hank’s solution (pH 7.2) for 1 to 2 min.

### 3.4. Cytotoxity of Streptozotocin (STZ)

Cells were seeded onto 96-well plates at a cell concentration of 1 × 10^5^ cells per well and were pre-incubated overnight. After pre-incubation, NIT-1 cells were exposed to toxin STZ. The cells were incubated for 24 h with or without a different concentration of STZ 10 μL (dissolved in 0.02 M acetate buffer) and the final concentrations were 0, 0.75, 1.5, 3, 6, 12 mM. Cytotoxicity of STZ on the NIT-1 cells was determined using MTT reduction assay.

### 3.5. MTT Assay

Cells were seeded at 1 × 10^5^ per well in a 96-well plate for viability assay. The media cultured with the cells were changed and MTT solution (5 mg/mL in PBS) 20 μL was added to each well and the plates were further incubated for another 6 h. Supernatants were then discarded and 150 μL of DMSO was added to the each incubation well and mixed thoroughly to dissolve the dark blue crystal formazan. The absorbance at 570 nm (formation of formazan) was recorded with a microplate spectrophotometer [22].

### 3.6. Experimental Design

Aliquots of 1 × 10^5^ NIT-1 cells were transferred into the wells of 96-well cell culture plates. After 48 h, STZ solution (final concentration 6 mM) was added to the each well of 96-well- plates and the cells were exposed to STZ for 24 h or were kept untreated as controls. Meanwhile, the cells were incubated for 24 h in the presence or absence of sesamin (dissolved in RPMI-1640 and the final concentration is 400, 200, 100 μg/mL). Immediately after cell culture, cell viability and condition were determined. Cell viability was determined by using a microtiter plate-based MTT assay, which has already been described. Cell condition was evaluated under the reverted microscope. Biochemical measurements of the supernatant and the harvested cells were also evaluated.

### 3.7. Microscopic Observation

Visual observations of NIT-1 cells under the inverted microscope were evaluated. The appearance of NIT-1 cells were observed under light microscope to evaluate the cell shape, the integrity of the cell membranes, confluence of the monolayer and the portion of dead cells.

### 3.8. Biochemical Measurements

The supernatants and the harvested cells in each well were collected and were used for biochemical assay. An estimation of levels of insulin secreted into the medium was assayed. The antioxidative capacity such as the activity of SOD and GSHpx, the content of GSH and MDA of NIT-1 cells were evaluated. The content of NO, the activity of NOS and iNOS of NIT-1 cells were also assayed. The above biochemical parameters were determined by using commercial kits according to the guidelines indicated. All samples were assayed in triplicate.

### 3.9. Statistical Analysis

Data were expressed as means ± SD Statistical analysis was evaluated by one-way analysis of variance, followed by the Student–Newman–Keuls test for multiple comparisons, which was used to evaluate the difference between two groups. *p* < 0.05 was considered significant.

## 4. Conclusions

Our study confirms that sesamin (SES) could protect pancreatic β cells damaged by streptozotocin (STZ) *in vitro*. The mechanisms may be related to its promoting cellular defense system by decreasing lipid per-oxidation and NO toxicity, increasing antioxidant enzyme activity, and, consequently, preserving the integrity and function of pancreatic β cells.

## Figures and Tables

**Table 1:** Effect of SES on cellular SOD, GSHpx, GSH and MDA in NIT-1 cells.

Group	Dose(μg mL^−1^)	SOD(U/mgpr)	GSHpx(U/mgpr)	GSH(mg/gpr)	MDA(nmol/mgpr)
Normal control	-	16.20 ± 2.38 ^*^	15.57 ± 2.00 ^**^	38.71 ± 4.92 ^*^	0.469 ± 0.061 ^**^
STZ model	-	11.42 ± 1.68	11.07 ± 1.44	30.82 ± 4.02	0.717 ± 0.128
SES	100	13.69 ± 1.56	13.17 ± 1.18 ^*^	35.65 ± 3.92	0.571 ± 0.079
	200	15.28 ± 1.30 ^**^	13.90 ± 1.53 ^*^	38.45 ± 4.42 ^*^	0.456 ± 0.063 ^**^
	400	16.54 ± 2.59 ^**^	14.68 ± 2.63 ^*^	40.06 ± 4.18 ^*^	0.458 ± 0.080 ^**^

Data are the mean ± SD (*n* = 10). ^*^p < *0.05* and ^**^p < 0.01 compared with STZ model control group.

**Table 2:** Effect of SES on cellular NO production by NIT-1 cells.

Group	Dose(μg mL^−1^)	NO(μmol/L)	NOS(U/mL)	iNOS(U/mL)
Normal control	-	56.94 ± 5.73 ^**^	1.92 ± 0.30 ^**^	0.64 ± 0.20 ^*^
STZ model	-	89.34 ± 5.12	3.01 ± 0.35	1.00 ± 0.23
SES	100	76.79 ± 4.42 ^**^	2.50 ± 0.25 ^*^	0.86 ± 0.28
	200	63.83 ± 5.26 ^**^	2.21 ± 0.35 ^*^	0.75 ± 0.30
	400	58.43 ± 3.31 ^**^	2.10 ± 0.18 ^**^	0.61 ± 0.20 ^*^

Data are the mean ± SD (*n* = 10). ^*^p < 0.05 and ^**^p < 0.01 compared with STZ model control group.
